# Association of increased pain intensity, daytime sleepiness, poor sleep quality, and quality of life with mobile phone overuse in patients with migraine: A multicenter, cross‐sectional comparative study

**DOI:** 10.1002/brb3.2760

**Published:** 2022-09-20

**Authors:** Mehwish Butt, Yeny Chavarria, Jesse Ninmol, Aabiya Arif, Sameer Saleem Tebha, Muhammad Daniyal, Umesa Mazhar Siddiqui, Syeda Samia Shams, Qubra Sarfaraz, Syeda Fatima Haider, Mohammad Yasir Essar

**Affiliations:** ^1^ Department of Neurosurgery and Neurology Jinnah Medical and Dental College Karachi Sindh Pakistan; ^2^ Department of Neurology University of California Irvine California; ^3^ Department of Public Health University of California Berkeley California; ^4^ Department of Medicine Ziauddin University Karachi Sindh Pakistan; ^5^ Department of Statistics The Islamia University of Bahawalpur Bahawalpur Punjab Pakistan; ^6^ Department of Research Kabul University of Medical Sciences Kabul Afghanistan

**Keywords:** daytime sleepiness, migraine, mobile phone overuse, quality of life, sleep quality

## Abstract

**Background**: The number of regular smartphone users has increased dramatically worldwide. Headaches, followed by sleep difficulties, forgetfulness, dizziness, and other ailments, are among the most prevalent complaints among smartphone users during or after use. In addition, migraine is a debilitating disease and is the world's second leading cause of disability. Hence, we performed this study to determine how smartphone overuse influenced migraine patients' level of disability, pain intensity, sleep quality, and overall quality of life.

**Methods**: In this observational study, the patients were divided into two groups high mobile phone use group (HMPUG) and the low mobile phone user group (LMPUG) using the Mobile Phone Problematic Use Scale. We assessed, for each group, patients’ level of disability, pain intensity, sleep quality, daytime sleepiness, and quality of life through the Migraine Disability Assessment Scale, Visual Analogue Scale, Pittsburgh Sleep Quality Index, Epworth Sleepiness Scale, and 24‐h Migraine Quality of Life Questionnaire, respectively.

**Results**: Our study showed that the respondents' average age was  27.59 (9.79) years. The average number of family members was 5.98 (2.3251). A total of 65.8% (*n* = 263) of the 400 participants were female, while 34.3 % (*n* = 137) were male. Greater pain intensity, poor sleep quality, and reduced medication effectivity were found in HMPUG compared to LMPUG (*p* < .05). However, increased duration of migraine and medication intake was reported in the LMPUG (*p* < .05).

**Conclusion**: We observed that smartphone overuse could worsen pain, sleep, and reduce treatment efficacy in individuals with migraine. Therefore, controlled smartphone use is recommended to avoid worsening symptoms.

## 1 INTRODUCTION

Since the development of the first smartphone of the modern era by Apple on January 9, 2007, there has been remarkable growth in the number of regular users throughout the world. By some estimates, around 6.648 billion people, about 83.72% of the world population, own and use a smartphone device (Smartphone users 2026, 2022). Furthermore, as the world rapidly becomes a virtual global village, smartphones are the foremost pertinent accelerators toward global digitalization (Montag & Diefenbach, 2018). With the increasing access of smartphones to a vast majority of the population, it has become crucial to assess and accurately calculate the adverse effects of this rampant technological advancement on its users.

Some of the most common complaints among users of smartphone devices are headaches followed by sleep disorders, forgetfulness, dizziness, etc., during or after smartphone use (Hocking & Westerman, 2002; Röösli et al., 2004). Literature has reported detailed clinical presentations of headaches secondary to smartphone usage (Demirci et al., 2016; Lee & Song, 2014). Furthermore, psychological, physical, and behavioral impairments include blurred vision, forgetfulness, pain in the hands and neck, and mood changes (Kwon et al., 2013). Although literature regarding smartphone usage and its effect on headaches is available, data specifically for migraines is relatively limited.

Migraine is a pervasive neurological disorder that, by some estimates, affects approximately 1 billion humans worldwide with a female predominance, and it is the second leading cause of disability and accounts for more disability than all other neurologic disorders combined (GBD 2016 Disease and Injury Incidence and Prevalence Collaborators, 2017; Stovner et al., 2016). From a global health perspective, it is crucial to know the effects of smartphone usage on the quality of life, sleep quality, and pain in migraine patients. As many as 175.62 million people in Pakistan are smartphone users, i.e., 79.50% of the total population of Pakistan (Smartphone Users, 2026, 2022). Furthermore, a 1‐year prevalence of migraine in Pakistan has been reported to be 22.9% by a study by Herekar et al. (2017). Thus, through our study, we hope to assess the frequency, intensity, and duration of headaches in patients suffering from migraine and their quality of life and sleep quality.

## 2 MATERIALS AND METHODS

### 2.1 Study design

This multicenter, cross‐sectional comparative study was conducted between August 2021 and January 2022 and was approved by the Institutional Review Board (IRB) of Jinnah Medical and Dental College (JMDC) and was assigned the protocol number 00094/21. The calculated sample size for this study was 270, with the anticipated frequency of people with migraine in Pakistan being 22.7% (Herekar et al., 2017). We recruited 400 smartphone users who visited the Neurology Clinic of two private hospitals and were diagnosed with migraine by a neurologist according to the diagnostic criteria of the International Classification of Headache Disorders‐III and clinical evaluation (Olesen, 2004). Patients between 18 and 65 who used their smartphones for more than 30 min each day and were free of any neurological problems in the previous 3 months were included. This study excluded patients with a history of any other neurological, psychiatric, or vascular disorders, and those who were hypertensive had coronary artery disease or were using any drugs as prophylaxis for migraine.

Our questionnaire included demographical questions, the Mobile Phone Problematic Use Scale (MPPUS) for identifying people in our sample with either low or high mobile phone use; the Visual Analogue Scale (VAS) for assessing pain severity; the Migraine Disability Assessment Scale (MIDAS) for determining the level of disability due to migraine; the 24‐h Migraine Quality of Life Questionnaire (24 h MQoLQ)] for assessing the quality of life; the Pittsburgh Sleep Quality Index (PSQI) for evaluating sleep quality, and the Epworth Sleepiness Scale (ESS) for assessing sleepiness in different scenarios (Ağargün et al., 1996; Buysse et al., 1989; Cline et al., 1992; Com.tr, 2022; Ertaş et al., 2004; Izci et al., 1999; Şar & Işıklar, 2012).

### 2.2 Mobile phone problematic use scale

MPPUS is a  scale used to measure the impact of mobile phone use frequency and overuse on social connections. In the literature, the MPPUS has also been employed by smartphone users (Van Deursen et al., 2015). The scale is divided into three sections: addiction (9 questions), social interactions (7 questions), and consequences (10 questions).  All items were graded on a scale of 0–4. The grading ranges from strongly disagree to strongly agree with the first two sections. In the final section of this scale, the grading ranges from not to very regularly. The level of smartphone use for each individual was assessed using an overall score, which runs from 0 to 104. This scale was used to create the low mobile phone use group (LMPUG) and high mobile phone use group (HMPUG). A score below the median, calculated for our sample, was classified as LMPUG, and a score above the median was classified as HMPUG (Izci et al., 1999; Şar & Işıklar, 2012).

### 2.3 Visual analogue scale

The VAS was used to evaluate pain intensity. The patient marks pain intensity on a 10‐cm ruler with zero being “no pain” and 10 being the “worst possible pain.” When compared to other one‐dimensional scales, VAS has been reported in the literature to be more sensitive and reliable in detecting pain severity (Cline et al., 1992).

### 2.4 Migraine disability assessment scale

The MIDAS assesses how headaches have affected the lives of patients diagnosed with migraine in the last 3 months. Patients respond to these questions by indicating the number of days they could not attend school/work, perform household chores, or be productive. Based on the responses, scores are calculated for the participants; the level of disability due to migraine is assessed using these scores, ranging from no disability due to migraine to severe disability (Ertaş et al., 2004).

### 2.5 24‐h Migraine Quality of Life Questionnaire

There are three components to the 24‐h Migraine Quality of Life Questionnaire, consisting of 15 items. These questions inquire about the symptoms, constraints on the person's life, and the psychological and social effects due to these symptoms caused by migraine. A higher overall score suggests a higher level of quality of life. In 2007, ltuş et al. assessed the questionnaire's cultural validity and reliability (Com.tr, 2022).

### 2.6 Pittsburgh sleep quality index

PSQI is used to assess total sleep duration, sleep quality, and sleep disorders during 1 month. Subjective sleep quality, sleep latency, sleep length, sleep activity, sleep disorders, medication usage, and daytime functioning are the seven components of this scale. The sum of all component scores yields the total PSQI score. A decline in sleep quality is indicated by a PSQI score greater than 5. Aargün et al. assessed the validity and reliability of this scale (Buysse et al., 1989; Ağargün et al., 1996).

### 2.7 Epworth sleepiness scale

The ESS is a tool that measures a person's general sleepiness throughout the day in eight different settings. The ESS employs a four‐point Likert scale. The scoring of this scale ranges from 0 to 24; depending on the score, the level of daytime sleepiness ranges from a “lower normal daytime sleepiness” to “severe excessive daytime sleepiness.” The ESS is a valid and reliable tool for determining levels of sleepiness (Izci et al., 1999).

### 2.8 Statistical analysis

Descriptive statistics were used to describe the sociodemographic and clinical characteristics of the study participant. Cronbach's alpha was calculated to measure the reliability of the questionnaire. Kolmogorov–Smirnov test was used to determine the normality of data. Mann–Whitney *U* test was used to compare the study variables. Multivariable linear regression was performed to assess determinants associated with mobile phone usage with the level of migraine. The data was analyzed using SPSS, version 22. The results were selected as statistically significant if the *p* ≤ .05.

## 3 RESULTS

Cronbach's alpha examined the reliability of the study. The overall reliability/consistency observed was good, with α = 0.798. A total of 400 participants participated in the study. The mean age of the respondents was 27.585(± 9.79) years. The average number of family members was 5.98(± 2.3251). Out of 400 participants, 65.8% (*n* = 263) were female and 34.3% (*n* = 137) were male respondents. There were 62.8 % (*n* = 251) participants who were unmarried and 37.3% (*n* = 149) were married. 23% (*n* = 92) of participants were Punjabi, 11.8% (*n* = 47) were Sindhi, 41.5% (*n* = 166) belonged to Muhajir ethnicity, and the rest were from other communities of the society. Considering the educational level of the respondents, 43.3% (*n* = 173) were graduate/postgraduate, 22% (*n* = 88) were pregraduate, and 13.8% (*n* = 55) were intermediate (Table 1).

**TABLE 1:** Demographic variables

Age	Mean ± SD	
	27.59 ± 9.79	
No. of family members (in figures)	Mean ± SD	
	5.98 ± 2.35	
Gender	*N*	%
Female	263	65.6
Male	137	34.2
**Educational status**		
Intermediate	55	13.7
Secondary	58	14.5
Primary	26	6.5
Pregraduate	88	21.9
Graduate/postgraduate	173	43.1
**Relationship status**		
Unmarried	251	62.6
Married	149	37.2

LMPUG and HMPUG have a mean age of 26.53(± 7.64) years and 28.64(± 11.48) years, respectively, significantly different with *p* = .031. The mean pain intensity in HMPUG is 5.88(± 2.51), which was significantly more than LMPUG, 5.11(± 2.77) with *p* = .009. The average frequency of migraine was 6.86 (± 5.89) in LMPUG and 6.57 (± 5.72) in HMPUG. This difference was not statistically significant, with *p* = .615. The presence of aura was reported significantly more by the LMPUG than in HMPUG with *p* = .000. LMPUG participants declared that they took medication to relieve the pain, which was more in numbers than HMPUG with *p* = .003. More participants in LMPUG declared relief due to medication in pain intensity and duration compared to HMPUG. This was only statistically significant for pain intensity with *p* = .007. 93.5% of the participants in LMPUG had episodic migraine, and 91.5% of participants in HMPUG had episodic migraine with no significant difference (*p* = .431) between the groups. More participants in HMPUG took Cafergot and Panadol for medication compared to LMPUG with a significant difference (*p* = .000) (Table 2).

**TABLE 2:** General attributes of the respondent's understudy

Attributes	Lower mobile phone usersGroup (LMPUG)	High mobile phone users group (HMPUG)	Test statistic	*p* Value
**Age (years)**				
Mean ± SD	26.537 ± 7.64	28.643 ± 11.489	–2.160	.031***
**Gender (%)**				
Male	70 (35.2)	67 (33.63)	0.151	.686
Female	129 (64.8)	134 (66.67)		
**Educational status**				
Intermediate	27 (13.4)	28 (14.1)	0.757	.108
Secondary	25 (12.4)	33 (16.6)		
Primary	11 (5.5)	15 (7.5)		
Pregraduate	38 (18.9)	50 (25.1)		
Postgraduate	100 (49.8)	73 (36.7)		
**Relationship status**				
Unmarried	122 (60.7)	129 (64.8)	0.729	.393
Married	79 (39.3)	70 (35.2)		
**No. of family members**				
Mean ± SD	5.79 ± 2.40	6.18 **±** 2.28	−0.168	.093
**The intensity of migraine pain**				
Mean ± SD	5.11 ± 2.77	5.88 ± 2.51	−2.642	.009***
**Frequency of migraine**				
Mean ± SD	6.86 ± 5.89	6.57 ± 5.72	0.504	.615
**Duration of migraine**				
Mean ± SD	1.91 ± 0.996	1.49 ± 0.758	4.720	.000***
**Presence of aura**				
Not present	166 (82.6)	184 (92.5)	31.206	.000***
Present	35 (17.4)	15 (7.5)		
**Any medication is taken to relieve pain**				
Yes	169 (84.1)	139 (69.8)	16.262	.003***
No	32 (15.9)	60 (31.2)		
**Does the medication reduce the pain intensity**				
Yes	17 (10)	25 (16.7)	9.794	.007***
No	17 (10)	25 (16.7)		
Sometimes	8 (4.7)	0 (0)		
**Does the medication reduce the duration of the migraine**				
Yes	115 (68)	108 (72)	1.367	.505
No	53 (31.4)	42 (28)		
Sometimes	1 (0.6)	0 (0)		
**Does the person have episodic or chronic migraine**				
episodic	188 (93.5)	182 (91.5)	0.621	.431
chronic	13 (6.5)	17 (8.5)		
**Type of medication used**				
Cafergot	32 (15.9)	49 (24.6)	34.374	.000***
Panadol	67 (33.4)	73 (38.2)		

***Significant if *p* < .05.

**FIGURE 1:**
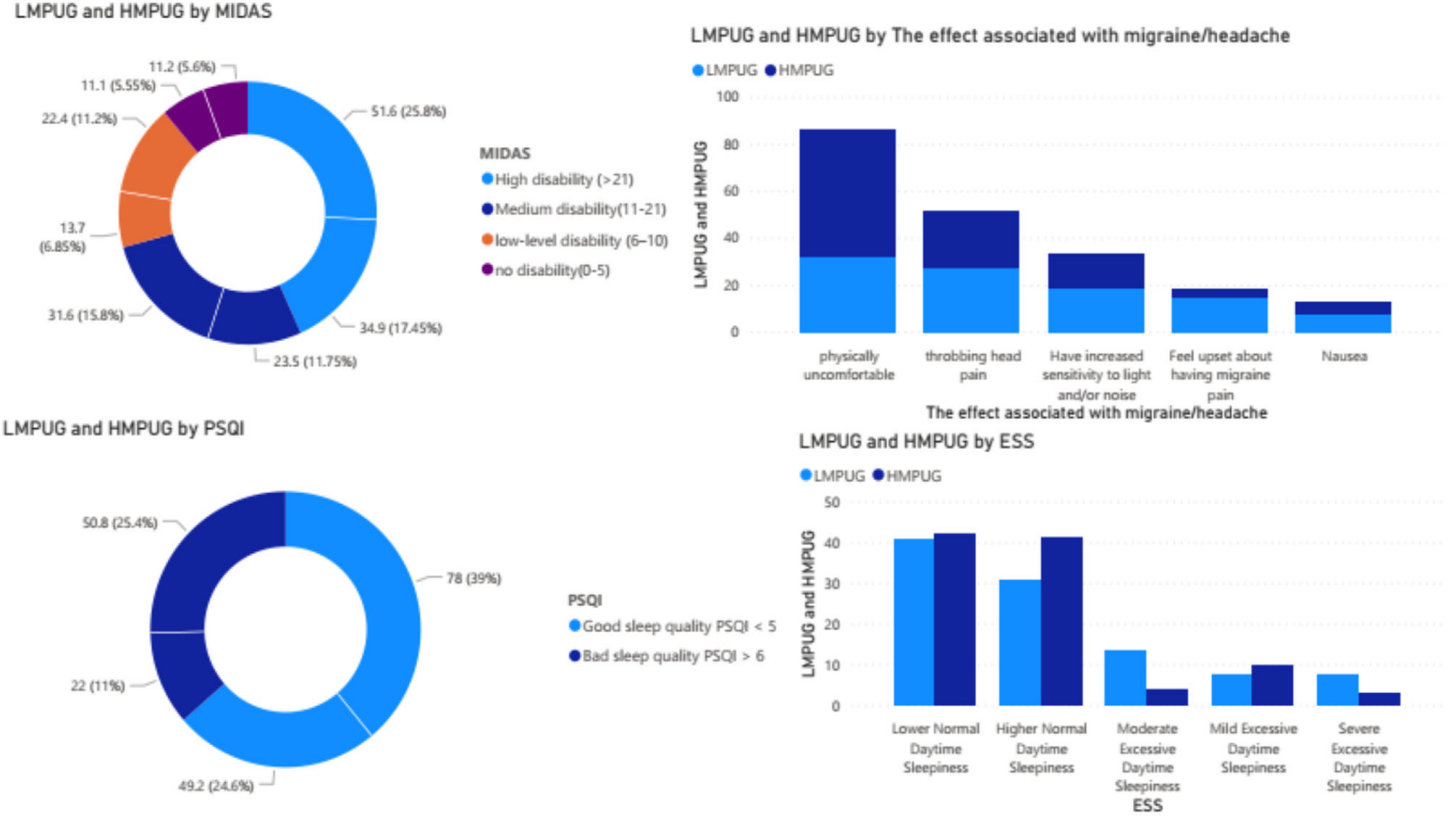
Frequency of different responses in MIDAS, ESS, PSQI, and migraine associated symptoms

As shown in Tables 3 and 4, it can be seen that the high disability level was more in LMPUG as compared to HMPUG, and there was a significant difference between the two groups with *p* = .019. 53.7% in HMPUG declared that they feel physically uncomfortable with migraine/headache, which was more than in LMPUG, and the difference was significant with *p* = .000. Bad sleep quality was found more in HMPUG than LMPUG, which PSQI assessed with a statistical difference at *p* = .000. According to ESS criteria, Higher Normal Daytime Sleepiness was observed more in HMPUG than those in LMPUG, and there is a significant difference between the two groups with *p* = .040. The comparison of outcomes has been compared using the Mann–Whitney *U* test, and it was observed that the pain intensity according to VAS was more in HMPUG (5.8 ± 2.5) than in LMPUG (5.1 ± 2.7), and there was a significant difference between these two with *p* = .009. According to quality of life, there was also a significant difference found with *p* = .000. Figure 1 summaries the frequency of responses from different scales.

**TABLE 3:** Comparison of the proportions between two groups (lower mobile phone users and high mobile phone users

Attributes of the study	Lower mobile phone users group	High mobile phone users group	Test statistic	*p* Value
**MIDAS**				
No disability (0–5)	17 (11.1)	17 (11.2)	9.905	.019***
Low‐level disability (6−10)	21 (13.7)	34 (22.4)		
Medium disability (11–21)	36 (23.5)	48 (31.6)		
High disability (>21)	79 (51.6)	53 (34.9)		
**The effect associated with migraine/headache**				
Have increased sensitivity to light and/or noise	37 (18.8)	28 (14.3)	62.878	.000***
Nausea	16 (8.1)	10 (5.1)		
Throbbing head pain	54 (27.4)	47 (24)		
Feel upset about having migraine pain	29 (14.7)	7 (3.6)		
physically uncomfortable	65 (32.3)	107 (53.7)		
**PSQI**				
Good sleep quality PSQI < 5	110 (78)	59 (49.2)	23.637	.000***
Bad sleep quality PSQI > 6	31 (22)	61 (50.8)		
**ESS**				
Lower normal daytime sleepiness	33 (40.7)	42 (42.0)	8.323	.040***
Higher normal daytime sleepiness	25 (30.9)	41 (41.0)		
Mild excessive daytime sleepiness	6 (7.4)	10 (10.0)		
Moderate excessive daytime sleepiness	11 (13.6)	4 (4.0)		

*Note*: Chi‐square Test of Association.

***Significant if *p* < .05.

**TABLE 4:** Comparison of outcomes between two groups

Factors	Low mobile phone user group	High mobile phone user group	*p* Value
**MIDAS**	22.6 ± 23.7	21.2 ± 23.0	.596
**PSQI**	66.5 ± 30.4	99.1 ± 209.9	.394
**ESS**	7.3 ± 4.9	6.0 ± 4.3	.067
**24‐MQoLQ**	38.4 ± 16.2	48.8 ± 22.1	.000***
**VAS**	5.1 ± 2.7	5.8 ± 2.5	.009***

*Note*: Mann–Whitney *U* test.

***Significant, if *p* < .05.

The Pearson's *r* correlation was calculated between the MPPUS and other scales to find the strength of association between them. A negative correlation was observed between MPPUS and MIDAS (*r* = −0.124), and this association was found significant at a level of 0.05 with *p* = .030. A positive correlation was found between MPPUS and PSQI with *r* = 0.301, and this relationship was significant (*p* = .000). MPPUS was also weakly negatively correlated with ESS with *r* = −0.136, but this relationship was significant with *p* = .041. There was a positive correlation between MPPUS and 24‐hMqolQ with *r* = 0.183. Their correlation was statistically significant with *p* = .000. VAS also found positively correlated with MPPUS with statistically significance (*r* = 0.131, *p* = .009). The details of the correlation are mentioned in Table 5.

**TABLE 5:** Correlation of MPPUS with outcome variables using Pearson's *r* correlation

MPPUS	Pearson's *r* correlation	*p* Value
**MIDAS**	–0.124	.030***
**PSQI**	0.301	.000***
**ESS**	–0.136	.041***
**24‐hMqolQ**	0.183	.000***
**VAS**	0.131	.009***

*Note*: Pearson's *r* correlation of MPPUS scale with MIDAS, PSQI, ESS, 24‐hMqolQ, and VAS. Significance level selected at .05.

***Significant if *p* < .05.

The relationship between quality of life has been examined with MPPUS, MIDAS, PSQI, ESS, and VAS. This relationship has been examined through binary logistic regression considering 24‐hMqolQ as the dependent variable, and other factors have been treated as IVs. The assumption of parallel lines was also tested, which confirmed the use of this technique and justified its preference over simple regression models.

$$\begin{array}{cc} \theta \left(Y=k|X=x_{mi}\right) & =\mathrm{logit}\varphi \left(x\right)=\ln\left[\frac{\varphi \left(x\right)}{1-\varphi \left(x\right)}\right]\\ & =\beta_{ok+\;}\beta_{1k\;}x_{1i}+\cdots +\beta_{nk\;}x_{ni}, \end{array}$$

where *Y* denotes the vector of dependent variables, and *X* denotes the vector for independent variables. The number of observations is given by *i* and *m* denotes the number of independent variables. The omnibus test of model coefficients is significant (*χ* ^2^ = 21.908, ***p = .000) showing that the model with these explanatory variables performs better in predicting the outcome. The following table has been obtained, which includes the coefficients (SE) with the *p* value. Quality of life has been positively estimated by VAS with a coefficient 0.319 (*p* = .007), negatively estimated by PSQI (β = −1.685 and *p* = .012**), ESS (β = −1.650, **p = .010), and MIDAS (β = −0.665, *p* = .013**). Quality of life was also positively dependent on MPPUS with a coefficient of 0.613 (*p* = .253). The coefficients with SE are mentioned in Table 6.

**TABLE 6:** Binary logistic regression coefficients (SE) with a *p* value

Predictors	β (SE)	*p* Value
**VAS**	0.319 (0.119)	.007***
**MPPUS**	0.613 (0.537)	.253
**PSQI**	–1.685 (0.670)	.012***
**ESS**	–1.650 (0.639)	.010***
**MIDAS**	–0.665 (0.268)	.013***

*Note*: Dependent variable: quality of life.

***Significant if *p* < .05.

## 4 DISCUSSION

With the widespread availability of smartphones, the rapid advancement in technology has drastically altered the patterns and amount of electronic media consumption profoundly. Smartphones have become ingrained in our professional, social, and personal lives. It has been reported that overuse of mobile phones can lead to multiple physical and psychological disorders. Hence, this study was conducted to understand better the interplay between overuse of mobile phones and pain severity, level of disability, sleep quality, daytime sleepiness, and quality of life in migraine sufferers.

Migraine is a debilitating disease; nearly half of the individuals suffering are unable to perform their daily tasks, owing to the severity of the disease. According to the AMPP (American Migraine Prevalence and Prevention) research, 48.2% of migraineurs had some level of disability, with 22.1% seriously impaired (Brandes, 2009). In our study, 33% of the individuals had a severe disability, while 34.8% reported some level of disability. However, this study observed a negative correlation between the MIDAS and MPPUS scales. This correlation was vindicated by other findings in our study, such as an increased level of disability and duration of migraine in the LMPUG compared to HMPUG. Contrary to our study, Yasmin Demir's study reported an increased duration and frequency of migraine in HMPUG but no difference in the level of disability between the two groups (Demir & Sumer, 2019). In addition, studies conducted in Iran, Egypt, and Poland have reported possible links between mobile phone use and headaches (Mortazavi et al., 2007; Salama & Abou El Naga, 2004; Szyjkowska et al., 2014). A study conducted by Agata Szyjkowska et al. (2014) reported that 26% of their participants experienced headaches that persisted longer than 6 h after mobile phone use. Various factors and triggers are associated with migraine, including but not limited to emotional stress, hormonal changes in women, light, poor posture, and smoking habits (Migraine causes, 2022). These and other factors could not be controlled in the studies; this could have influenced contrasting findings. However, the degree of pain in HMPUG was considerably higher than that of LMPUG in the current and Yasmin Demir's study (Demir & Sumer, 2019). Conversely,  the study done by Uttarwar et al. (2020) reported no significant difference in the pain intensity and headaches between smartphone users and non‐smartphone users. Even though LMPUG had increased the number of medications taken, HMPUG reported not feeling relieved in pain upon taking medication more than the LMPUG. Uttarwar et al. (2020) also reported medication being less effective among smartphone users.

The suggested etiology in the literature for headaches caused by mobile phone use includes the posture changes producing strain on the cervical spine, blue light exposure from the screen leading to strain, heat, sound, and visual stimulus from the mobile phones, and the most controversial factor being electromagnetic radiations (EMR). It is thought that the EMR disrupts neurotransmission, the brain's electrical activity, and the blood–brain barrier (Chu et al., 2011; EMF: 4, 2022). The meta‐analysis conducted in 2012 revealed no association between EMR exposure and headaches (Augner et al., 2012). However, the most recent meta‐analysis conducted by Farashi et al. (2022) reported an association between headaches and EMR exposure in both the young and the old.

Moreover, multiple studies have found that EMR also harms sleep quality. Smartphone users have trouble falling and staying asleep, and their quality of life suffers due to their use. Sarah Loughran et al. (2005) found that a 30‐min exposure to electromagnetic radiation before sleep increased rapid eye movement sleep and caused alterations in the electroencephalogram during the non‐rapid eye movement phase of sleep. The blue light emission by cell phone screens also negatively influences sleep quality (Elhai et al., 2017). Hence, it was not surprising that HMPUG had poorer sleep quality than LMPUG, which was assessed using a PSQI scale. Moreover, a positive correlation between MPPUS and PSQI was observed in our study. Although the ESS scale reported greater daytime sleepiness experienced by the LMPUG, a negative correlation was also observed between the ESS scale and the MPPUS. The reason for these incongruent findings regarding sleep could be explained by the self‐administered, hence subjective nature of this scale. Simultaneously, other factors like caffeine intake that can affect the level of daytime sleepiness experienced by the individuals were not taken into account. The study conducted by Demir and Sumer (2019) promulgated conflicting results to ours by reporting a negative correlation between the MPPUS and PSQI and a positive correlation between MPPUS and ESS.

Inadequate sleep may wreak havoc on one's mental health and predisposes one to disorders like depression and anxiety (Demirci et al., 2015). In addition, poor sleep quality is associated with overall poor quality of life (Demir & Sumer, 2019; Lee et al., 2021). Similarly, an inverse correlation was observed between the sleep scales employed in this study (ESS and PSQI) and 24‐hMqolQ. Withal, a negative correlation was also observed between the MPPUS and 24‐hMqolQ, which translates to increased quality of life with increased mobile phone usage. A possible explanation for this finding could be that the individuals with increased phone usage spend more time socializing on their phones, which could delineate that they have a wider social circle and social support; conjointly, these individuals might also be spending more time on their smartphones to relax. All these factors can directly or indirectly contribute to a better quality of life.

The strength of this multicenter study is a large sample size, with a balanced distribution between LMPUG and HPMUG. Thus, the findings of this study are generalizable. The limitations of this study include the self‐reporting nature of all the scales used; hence the data are subjected to recall bias. In addition, various factors can influence and trigger migraine in individuals, some not even quantifiable; these confounders are a limitation of this study.

The present literature is still limited, and further research is needed to comprehend the interplay between smartphone use and migraines completely. In addition, studies with a prospective study design and controlled smartphone exposure and environmental conditions are needed to determine the causal link.

## 5 CONCLUSION

We conclude that migraineurs' excessive use of mobile phones leads to increased pain intensity, reduced medication effectivity, and poor sleep quality. However, greater mobile phone use does not lead to increased disability, duration, and frequency of migraines and is associated with better quality of life. Further research is required to understand the mechanism underlying smartphone usage and its negative consequences, and effective treatment must be identified for these individuals.

## FUNDING

No funding was received to assist with the preparation of this manuscript.

## FINANCIAL INTEREST

The authors have no relevant financial or nonfinancial interest to disclose

## INFORMED CONSENT

Written informed consent was acquired from all participants for collection or relevant data as well as publication of it for academic and research purposes.

## AUTHOR CONTRIBUTIONS

All authors contributed to the study conception and design. Material preparation, data collection, and analysis were performed by Aabiya Arif, Sameer Saleem Tebha, Muhammad Daniyal, Umesa Mazhar Siddiqui, and Syeda Samia Shams. The first draft of the manuscript was written by Mehwish Butt, Yeny Chavarria, Jesse Ninmol, Syeda Fatima Haider, Qubra Sarfaraz, and Mohammad Yasir Essar, and all authors commented on previous versions of the manuscript. All authors read and approved the final manuscript.

## STATEMENT OF ETHICS

This study was approved by the Institutional Review Board (IRB) of Jinnah Medical and Dental College (JMDC) and was assigned the protocol number 00094/21.

## Data Availability

All relevant data are part of the manuscript, but if any other information is required, it can be inquired upon to the corresponding author.
